# Impact of hemodialysis and post-dialysis period on granular activity levels

**DOI:** 10.1186/s12882-020-01853-2

**Published:** 2020-05-25

**Authors:** John W. Larkin, Maggie Han, Hao Han, Murilo H. Guedes, Priscila Bezerra Gonçalves, Carlos Eduardo Poli-de-Figueiredo, Américo Lourenço Cuvello-Neto, Ana Beatriz L. Barra, Thyago Proença de Moraes, Len A. Usvyat, Peter Kotanko, Maria Eugenia F. Canziani, Jochen G. Raimann, Roberto Pecoits-Filho, For the HDFIT Study Investigators, Ana Claudia Dambiski, Thaylane Amanda de Souza, Daniela Ponce, Edwa Maria Bucuvic, Luciana Menin Ferreira, Wanderson de Souza Carvalho, Jorge Paulo Strogoff de Matos, Esther Oliveria Silva, Manuel Carlos Martins de Castro, Celina de Fátima e Silva, Maria Eugenia F. Canziani, Silvia R. Manfredi, Katia Santos, Ana Paula Fonseca Correia, Giovani Gadonski, Adriana Conti, Inah Pecly, Camille Souza Paixão, Viviane Calice-Silva, Simone Ribeiro, Lizia Regina Ribeiro Caldeira, Adailto Santos, Rosilene Motta Elias, Andreia Barbosa Dos Santos, Américo Lourenço Cuvello-Neto, Amanda Monteiro Virolli

**Affiliations:** 1 School of Medicine, Pontifícia Universidade Católica do Paraná, Imaculada Conceição, 1155, Curitiba, PR 80215-901 Brazil; 2 Global Medical Office, Fresenius Medical Care, Waltham, MA USA; 3 Research Division, Renal Research Institute, New York, NY USA; 4 Health Technology Graduate Program, Pontifícia Universidade Católica do Paraná, Curitiba, PR Brazil; 5 School of Medicine, Pontifícia Universidade Católica do Rio Grande do Sul, Porto Alegre, RS Brazil; 6 Hospital Alemão Oswaldo Cruz, São Paulo, SP Brazil; 7 Fresenius Medical Care Brazil, São Paulo, SP Brazil; 8 Icahn School of Medicine at Mount Sinai, New York, NY USA; 9 Universidade Federal de São Paulo, São Paulo, SP Brazil

**Keywords:** High-flux hemodialysis, Physical activity, Steps, Moderate-to-vigorous activity, Accelerometry, End stage kidney disease

## Abstract

**Background:**

Physical activity (PA) is typically lower on hemodialysis (HD) days. Albeit intradialytic inactivity is expected, it is unknown whether recovery after HD contributes to low PA. We investigated the impact of HD and post-HD period on granular PA relative to HD timing.

**Methods:**

We used baseline data from the HDFIT trial conducted from August 2016 to October 2017. Accelerometry measured PA over 1 week in patients who received thrice-weekly high-flux HD (vintage 3 to 24 months), were clinically stable, and had no ambulatory limitations. PA was assessed on HD days (0 to ≤24 h after start HD), first non-HD days (> 24 to ≤48 h after start HD) and second non-HD day (> 48 to ≤72 h after start HD). PA was recorded in blocks/slices: 4 h during HD, 0 to ≤2 h post-HD (30 min slices), and > 2 to ≤20 h post-HD (4.5 h slices). Blocks/slices of PA were captured at concurrent/parallel times on first/second non-HD days compared to HD days.

**Results:**

Among 195 patients (mean age 53 ± 15 years, 71% male), step counts per 24-h were 3919 ± 2899 on HD days, 5308 ± 3131 on first non-HD days (*p* < 0.001), and 4926 ± 3413 on second non-HD days (*p* = 0.032). During concurrent/parallel times to HD on first and second non-HD days, patients took 1308 and 1128 more steps (both *p* < 0.001). Patients took 276 more steps and had highest rates of steps/hour 2-h post-HD versus same times on first non-HD days (all *p* < 0.05). Consistent findings were observed on second non-HD days.

**Conclusions:**

PA was higher within 2-h of HD versus same times on non-HD days. Lower PA on HD days was attributable to intradialytic inactivity. The established PA profiles are of importance to the design and development of exercise programs that aim to increase activity during and between HD treatments.

**Trial registration:**

HDFIT was prospectively registered 20 April 2016 on ClinicalTrials.gov (NCT02787161)

## Background

A physically active lifestyle can improve and maintain health in children, adults, and populations with disabilities or chronic diseases [1–4]. People with end stage kidney disease (ESKD) typically perform low levels of physical activity (PA) (e.g. < 5000 steps/day), placing them at higher risk for obesity, diminished quality of life, cardiovascular diseases, and mortality [3–8]. Inactivity in ESKD is likely influenced by high prevalence of physical disabilities and comorbidities, and social, environmental, and patient characteristics [3, 4, 9–15]. Albeit lifesaving, dialysis can be associated with symptoms such as nausea, body aches, and fatigue impacting PA [12, 16, 17]. Understanding profiles and drivers of PA in ESKD is important to find optimal treatment options and design interventions.

Profiles of objectively measured PA are loosely defined in ESKD. Studies in hemodialysis (HD) populations using pedometers/accelerometers report average PA levels range from 2446 to 8454 steps/day; PA is lower on HD days versus non-HD days [6, 7, 18–25]. and PA decreases with longer vintage [18, 24]. A limitation of most studies conducted to date is they assessed PA by calendar days (i.e. 0:00–23:59 h) and have not assessed PA relative to the timing of HD [2, 5–7, 18–27]. It is unknown if recovery after HD may influence PA on HD days in addition to requirements of sitting/lying during HD. With the potential impacts of HD on PA, granular assessments are needed to find timepoints for risk assessment and interventions.

PA was measured via accelerometry in HD patients during the baseline period of the HDFIT randomized controlled trial (RCT) [28, 29]. Various types of PA were assessed in 24-h periods relative to the start of HD (0-to- ≤ 24 h after start HD) and concurrent/parallel periods to HD days on the subsequent first and second non-HD days (> 24-to- ≤ 48 and > 48-to- ≤ 72 h after start HD, respectively). PA data was captured in granular slices. Our primary hypothesis was granular slices of PA would be lower on HD days, particularly immediately after HD, versus concurrent time segments on the first and second non-HD days.

## Methods

### Trial design

HDFIT was a prospective, multi-center, unblinded, RCT studying the impact of modality on objective PA [28]. The HDFIT protocol and trial design has been previously published [29]. After a run-in baseline period up to 4 weeks, eligible participants were randomized in a 1:1 ratio to high-volume online hemodiafiltration (HDF) or high-flux HD for a 6-month interventional period and 12-month observational follow-up. This ancillary study investigated PA profiles during the run-in baseline period (ClinicalTrials.gov Identifier: NCT02787161; registration date 20 April 2016).

### Setting and participants

Fourteen dialysis centers in Brazil were activated for recruitment. The Center for Epidemiology and Clinical Research (EPICENTER) academic clinical research organization (ACRO) based at Pontifícia Universidade Católica do Paraná (PUCPR) managed the trial.

Patients provided written informed consent before study activities. Trial included adults (age ≥ 18 years) who initiated renal replacement therapy between 3 and 24 months prior to randomization, were treated with thrice-weekly HD, used a fistula/graft or permanent catheter, had previous Kt/V ≥ 1.2, and were considered clinically stable. Patients with severely limited mobility/ambulation, poor treatment adherence, or a life expectancy of < 3 months because of non-renal comorbidities were excluded.

### Ethical considerations

The protocol, consent form, and study documents were approved PUCPR ethics review board (central application# 54926916.7.1001.0020; approval# 1.538.784). Trial was conducted in accordance with the Declaration of Helsinki.

### Procedures and interventions

PA was continuously measured over 7 days using a validated waist worn tri-axial accelerometer (ActiGraph™ wGT3X-BT model, Pensacola, USA) [19, 30–33]. Patients were instructed to remove the accelerometer during sleep and bathing, and record their HD, sleep, and bathing times during monitoring.

Patient demographics (age, sex, race), clinical, laboratory, and social parameters were recorded from medical records and/or assessments. Clinical characteristics applicable to this ancillary study included etiology of ESKD, comorbidities, access type, height, pre-HD/post-HD/estimated dry weight, pre-HD/post-HD systolic and diastolic blood pressure (SBP/DBP), pulse, and HD parameters. Educational level, family income, employment status, transportation method to clinic, and distance from clinic to the patient’s residence was also captured.

Recent monthly values for pre−/post-HD blood urea nitrogen (BUN) and hemoglobin, and quarterly values for albumin, potassium, calcium, phosphate, intact parathyroid hormone (iPTH) were captured from the medical records. HD adequacy (single-pool Kt/V) was calculated from BUN [34].

### Endpoints

Primary endpoints were the difference in steps and moderate-to-vigorous PA (MVPA) per 24 h and between prespecified periods after dialysis on HD days versus concurrent/parallel periods at the same time on subsequent first or second non-HD days; similar assessments were performed for light PA, sedentary time, and metabolic rate (MET) levels. Secondary endpoints were the difference in steps and MVPA per 24 h and in prespecified periods after the start of dialysis: i) on the first versus second or third HD session of the week, and ii) HD start before versus after 1500 h. A further exploratory analysis of the difference in sedentary time per 24 h and in prespecified periods was performed in patients starting HD before versus after 1500 h.

An exploratory analysis was performed for a sub-group assessment of the difference in step counts and sedentary time in the prespecified periods 24 h after the start of HD stratified by transportation category for public (use of public transportation or walking) versus car (use of a family car, taxi, or ambulance) transportation. Also, sub-group analysis was performed to calculate the difference in step counts and sedentary time per 24 h after HD initiation by HD shifts starting before/after 1500 h stratified by age category (< 65 versus ≥65 years old) and family income category (< 2 versus > 2 minimum wages).

### Statistical methods

#### Physical activity analysis

Raw accelerometer data were uncompressed using ActiLife v6.13.3 software (ActiGraph LLC, Pensacola, USA) and bandpass filtered using default filter with a 60 s epoch length. Data validation and filtering of non-wear time measurements was performed via vector magnitude and Choi 2011 algorithm [35]. We used a 60-min window for consecutive zeros/non-zeros [36].

PA data was cleaned, sliced, computed and exported using a custom coded file denoting slices based off start and end time of each HD session. Freedson VM3 Combination (2011) algorithm was used to calculate energy expenditure [37]. Raw acceleration cut points were: Sedentary = 0–99 cpm (CPM); Light = 100–2689 CPM; Moderate =2690–6166 CPM; Vigorous = 6167–9642 CPM; Very Vigorous = 9643-infinity CPM [37]. Freedson Adult 1998 algorithm was used to calculate MET levels [38]. MET levels represent the amount of energy expended per unit time in reference to sitting/resting MET level of 1. If monitor was worn during sleep, custom export files were coded to exclude the data using patient-reported sleep times.

**Fig. 1:**
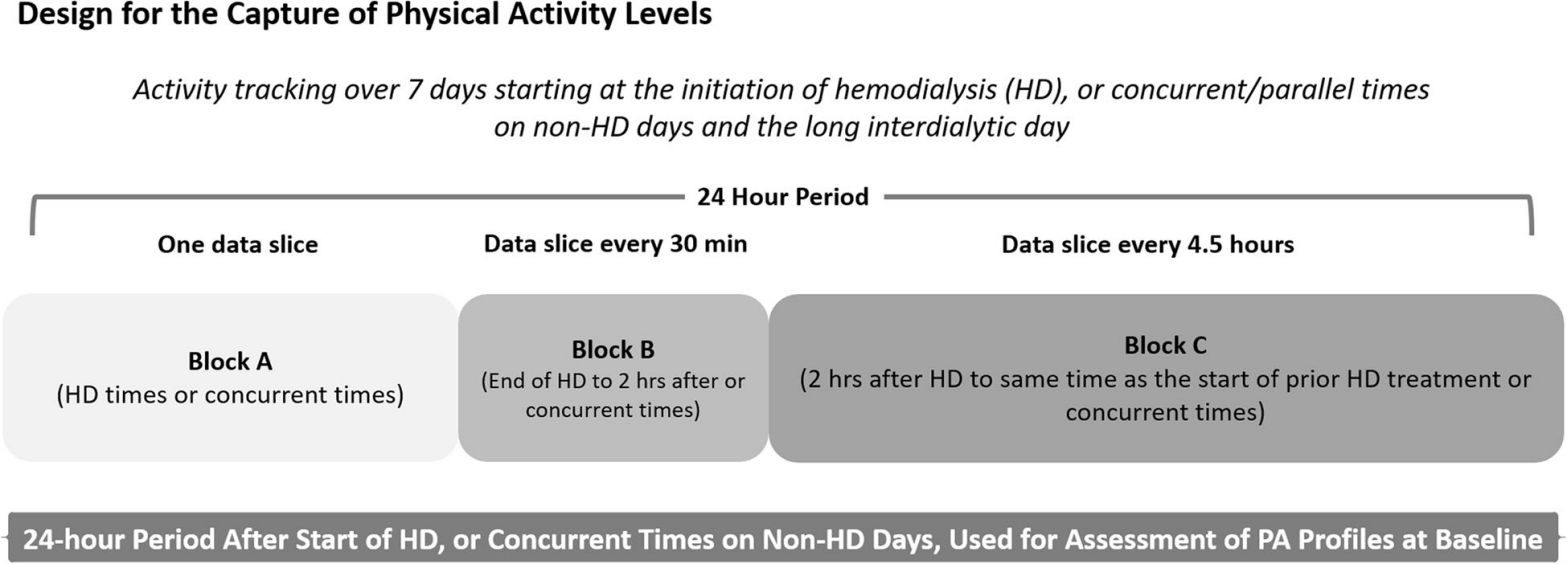
Schematic of the design for the export and analysis of physical activity levels in in reference to the timing of the start of HD, or concurrent/parallel times on non-HD days. Data was capture over 24 h periods in 3 blocks. The blocks were sliced to periods to investigate granular profiles after HD

Extraction of 24-h periods of PA data was performed in 3 blocks consisting of 9 slices: Block A) HD/concurrent non-HD period with 1 slice of PA data, Block B) ≤2-h post-HD/concurrent non-HD period with four data slices of 30 min each, and Block C) > 2-to- ≤ 20-h post-HD/concurrent non-HD period with four data slices of 4.5 h each (Fig. 1).

#### Analysis of endpoints

Primary endpoints were analyzed by ANOVA and pooled two-way t-tests. Granular PA data for steps and minutes of MVPA was normalized to a rate per hour metric for visualization of data in prespecified periods. Secondary endpoints (i) and (ii) were compared using paired and unpaired t-tests, respectively.

Exploratory sub-group analysis of step counts and sedentary time in prespecified periods on HD days by transportation type category were compared using unpaired t-tests. Comparisons for sub-group analysis of steps counts and sedentary time per 24 h on HD days in HD shifts starting before/after 1500 h by age and family income categories were performed with unpaired t-tests.

## Results

### Patient characteristics

**Fig. 2:**
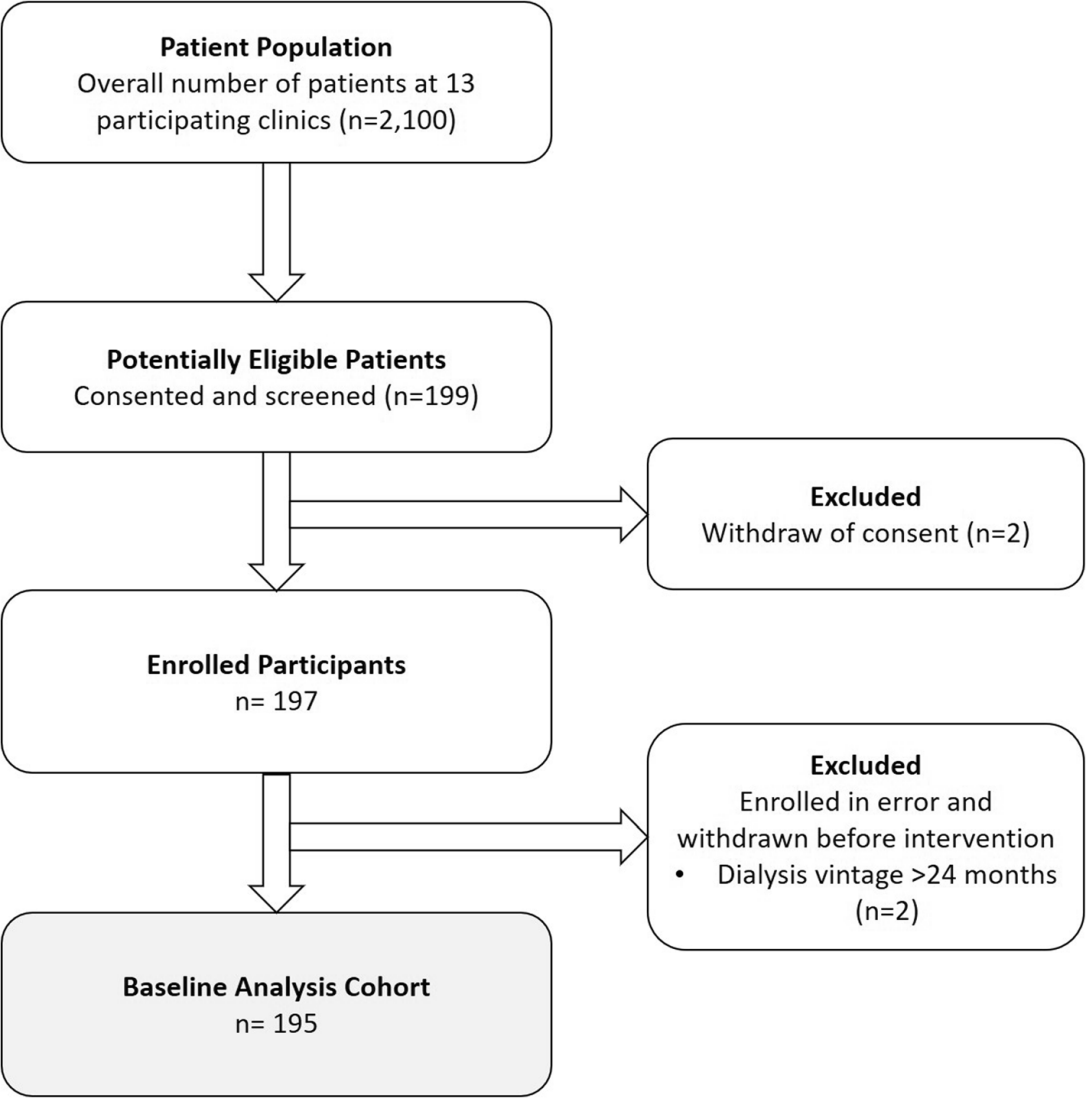
Participant Flow Diagram

HDFIT enrolled 195 patients from 13 of 14 sites throughout Brazil during August 2016 to October 2017 (Fig. 2). On average, patients were 53 years old, 71% male, 59% white race, 35% had diabetes, 17% had coronary artery disease, 8% had congestive heart failure; 89% used an arteriovenous fistula/graft and 55% had ESKD attributable to diabetes/hypertension. Family income was 2-to-10 times the Brazilian minimum wage in 73% and below that range in 18%. Fifty-six percent completed high school or higher. Seventy-two percent resided ≥7 km from the dialysis clinic; 43% drove by car and 33% by public transportation to the HD clinic. Mean albumin was 4.0 ± 0.4 g/dL, phosphate 5.3 ± 1.4 mg/dL, iPTH 350.5 ± 290.0 pg/mL, hemoglobin 11.1 ± 1.6 g/dL, and Kt/V 1.5 ± 0.4 (Table 1).

**Table 1:** Patient Characteristics

Parameter	Category	Total N	Mean (±SD) or N and %
**Age (years)**		195	52.98 (±15.08)
**Male (%)**		195	139 (71)
**Race**	*White (%)*	195	115 (59)
	*Other (%)*		80 (41)
**Comorbidities**	*Diabetes (%)*	195	68 (35)
	*Coronary artery disease (%)*		33 (17)
	*Congestive heart failure (%)*		15 (8)
**Dialysis access type**	*Arteriovenous fistula (%)*	195	164 (84)
	*Arteriovenous graft (%)*		9 (5)
	*Permanent catheter (%)*		22 (11)
**Etiology of CKD/ESKD**	*Diabetic nephropathy (%)*	195	56 (29)
	*Hypertensive nephrosclerosis (%)*		51 (26)
	*Chronic glomerulonephritis (%)*		32 (16)
	*Interstitial nephritis (%)*		7 (4)
	*Chronic pyelonephritis (%)*		7 (4)
	*Polycystic Kidney Disease (%)*		11 (6)
	*Lupus Nephritis (%)*		5 (3)
	*Other (%)*		11 (6)
	*Unknown (%)*		15 (8)
**Family income level**	*Above 10 minimum wages (%)*	195	17 (9)
	*4 to 10 minimum wages (%)*		54 (28)
	*2 to 4 minimum wages (%)*		88 (45)
	*Less than 2 minimum wages (%)*		36 (18)
**Education level**	*Complete university (%)*	195	39 (20)
	*Complete high school (%)*		70 (36)
	*Complete elementary (%)*		53 (27)
	*Incomplete elementary (%)*		33 (17)
**Distance from home to clinic**	*Greater than 25 Km (%)*	195	48 (25)
	*Between 13 and 25 Km (%)*		47 (24)
	*Between 7 and 13 Km (%)*		46 (24)
	*Less than 7 Km (%)*		52 (27)
	*Missing (%)*		2 (1)
**Transportation type to clinic**	*Family car (%)*	195	84 (43)
	*Public transportation (%)*		65 (33)
	*Ambulance (%)*		32 (16)
	*Taxi (%)*		9 (5)
	*Walking (%)*		5 (3)
**Height (cm)**		195	168.13 (±8.42)
**Estimated dry weight (Kg)**		194	75.25 (±15.92)
**Pre-dialysis weight (kg)**		194	77.78 (±15.98)
**Post-dialysis weight (kg)**		193	75.51 (±15.79)
**Pre-dialysis SBP (mmHg)**		193	153.19 (±23.7)
**Pre-dialysis DBP (mmHg)**		193	80.93 (±13.43)
**Pre-dialysis pulse (beats per minute)**		194	75.58 (±12.61)
**Post-dialysis SBP (mmHg)**		192	148.14 (±23.01)
**Post-dialysis DBP (mmHg)**		190	77.44 (±13.37)
**Post-dialysis pulse (beats per minute)**		189	74.39 (±11.93)
**Laboratories**	*Pre-HD BUN (mg/dL)*	189	58.2 (13.1)
	*Post-HD BUN (mg/dL)*	195	17.1 (7.7)
	*Kt/V*	194	1.5 (0.4)
	*Albumin (g/dL)*	192	4.0 (0.4)
	*Potassium (mEq/L)*	195	5.2 (0.8)
	*Calcium (mg/dL)*	175	9.0 (0.7)
	*Phosphate (mg/dL)*	195	5.3 (1.4)
	*Intact parathyroid hormone (pg/mL)*	190	350.5 (290.0)
	*Hemoglobin (g/dL)*	193	11.1 (1.6)

Descriptive statistics are presented by parameter and category as applicable. The number of patients with valid data for each parameter are detailed in the third column (Total N); missing category data is detailed as applicable. The descriptive statistics in the fourth column are presented as the mean value ± standard deviation (SD) for continuous variables or as the patient number (N) and percent (%) for categorical variables

### Profiles of physical activity

Among 195 HD patients with PA monitored, 176 had valid data. On average, patients took 4654 ± 3468 steps, their energy expenditure rate was 1.1 ± 0.2 MET (kcal/kg/hr), and they performed 23.9 ± 32.6 min of MVPA, 361.2 ± 134.5 min of light PA, and 488.0 ± 148.0 min of sedentary time per 24-h period (Table 2). ANOVA of PA on HD days and the first, and second non-HD days revealed differences between groups for steps (*p* < 0.001), MET (*p* = 0.043), MVPA (*p* < 0.001), light PA (*p* < 0.0001) and sedentary time (*p* < 0.001) per 24 h.

**Table 2:** Average Physical Activity (PA) Levels Per 24 Hours Overall and on HD Days, the First Non-HD Days and Second Non-HD Day

PA Measure (*N* = 176)	Overall (±SD)	HD Day (±SD)	1st Non-HD Day (±SD)	***P***-Value vs HD Day	2nd Non-HD Day (±SD)	***P***-Value vs HD Day
	***Per 24 Hrs***	***0-to- ≤ 24 Hrs after Start HD***	***> 24-to- ≤ 48 Hrs after Start HD***		***> 48-to- ≤ 72 Hrs after Start HD***	
Steps Count	4654 (3468)	3919 (2899)	5308 (3131)	< 0.001	4926 (3413)	0.032
MET (kcal/kg/Hr)	1.08 (0.19)	1.09 (0.17)	1.09 (0.23)	0.797	1.07 (0.18)	0.027
MVPA Minutes	23.9 (32.6)	20.3 (27.0)	27.5 (31.3)	0.021	24.3 (28.3)	0.175
Light PA Minutes	361.2 (134.5)	294.5 (95.5)	402.5 (127.3)	< 0.0001	386.7 (149.5)	< 0.0001
Sedentary Time Minutes	488.0 (148.0)	523.2 (118.2)	453.4 (136.3)	< 0.0001	487.3 (176.2)	0.026

On the first non-HD days, patients performed 1389 more steps (*p* = 0.032), 7.2 more minutes of MVPA (*p* = 0.021), 107.9 more minutes of light PA (*p* < 0.0001), and 69.8 fewer minutes of sedentary time (*p* < 0.0001) per 24-h compared to HD days. MET levels were the same on the first non-HD days versus HD days (Table 2).

On the second non-HD day, patients performed 1007 more steps (*p* < 0.001), 0.02 lower MET (*p* = 0.027), 92.2 more minutes of light PA (*p* < 0.0001), and 35.8 fewer minutes of sedentary time (*p* = 0.026) per 24-h compared to HD days. Minutes of MVPA were similar for the second non-HD day versus HD days (Table 2).

### Granular profiles of physical activity

Assessment of granular PA levels revealed, as expected, that sitting/lying during HD was associated with the most remarkable differences on HD days versus concurrent/parallel times on non-HD days. There were small increases in PA during the 2-h post-HD period versus the same times on non-HD days (Table 3).

**Table 3:** Average Physical Activity (PA) Levels Per Predefined Granular Slice Period on HD Days, the First Non-HD Days and Second Non-HD Day

Step Counts Per Predefined Granular Slice Period (***N*** = 176)			
**Slice Period during/after HD or Concurrent Time**	**HD Day (±SD)**	**1st Non-HD Day (±SD)**	**2nd Non-HD Day (±SD)**
	***0-to- ≤ 24 Hrs after Start HD***	***> 24-to- ≤ 48 Hrs after Start HD***	***> 48-to- ≤ 72 Hrs after Start HD***
HD Period	97 (225)	1405*** (1067)	1225*** (1468)
0.0- ≤ 0.5 Hr Post-HD	261 (267)	180** (179)	137*** (177)
> 0.5- ≤ 1.0 Hr Post-HD	282 (224)	203** (299)	147*** (211)
> 1.0- ≤ 1.5 Hr Post-HD	243 (200)	164*** (183)	173* (323)
> 1.5- ≤ 2.0 Hr Post-HD	179 (181)	142* (149)	148 (218)
> 2.0- ≤ 6.5 Hr Post-HD	788 (772)	941 (925)	844 (926)
> 6.5- ≤ 11.0 Hr Post-HD	608 (729)	718 (926)	704 (1058)
> 11.0- ≤ 15.5 Hr Post-HD	964 (1668)	874 (1343)	1014 (1749)
> 15.5- ≤ 20.0 Hr Post-HD	1257 (1352)	1327 (950)	1384 (1082)
**MET Rate Per Predefined Granular Slice Period (*N*** **= 176)**			
**Slice Period during/after HD or Concurrent Time**	**HD Day (±SD)**	**1st Non-HD Day (±SD)**	**2nd Non-HD Day (±SD)**
	***0-to- ≤ 24 Hrs after Start HD***	***> 24-to- ≤ 48 Hrs after Start HD***	***> 48-to- ≤ 72 Hrs after Start HD***
HD Period	1.0 (0.03)	1.1*** (0.2)	1.1*** (0.2)
0.0- ≤ 0.5 Hr Post-HD	1.1 (0.3)	1.1 (0.3)	1.0*** (0.1)
> 0.5- ≤ 1.0 Hr Post-HD	1.1 (0.2)	1.1 (0.4)	1.0*** (0.2)
> 1.0- ≤ 1.5 Hr Post-HD	1.1 (0.2)	1.1 (0.2)	1.1 (0.3)
> 1.5- ≤ 2.0 Hr Post-HD	1.1 (0.2)	1.1 (0.2)	1.1 (0.2)
> 2.0- ≤ 6.5 Hr Post-HD	1.1 (0.1)	1.1 (0.3)	1.0 (0.1)
> 6.5- ≤ 11.0 Hr Post-HD	1.1 (0.2)	1.1 (0.2)	1.1 (0.2)
> 11.0- ≤ 15.5 Hr Post-HD	1.1 (0.2)	1.1 (0.2)	1.1 (0.3)
> 15.5- ≤ 20.0 Hr Post-HD	1.1 (0.1)	1.1 (0.1)	1.1 (0.1)
**Minutes of MVPA Per Predefined Granular Slice Period (*N*** **= 176)**			
**Slice Period during/after HD or Concurrent Time**	**HD Day (±SD)**	**1st Non-HD Day (±SD)**	**2nd Non-HD Day (±SD)**
	***0-to- ≤ 24 Hrs after Start HD***	***> 24-to- ≤ 48 Hrs after Start HD***	***> 48-to- ≤ 72 Hrs after Start HD***
HD Period	0.3 (10.8)	7.5*** (1.6)	5.8*** (12.4)
0.0- ≤ 0.5 Hr Post-HD	1.3 (1.8)	0.9 (2.4)	0.6** (1.7)
> 0.5- ≤ 1.0 Hr Post-HD	1.2 (2.6)	1.1 (2.0)	0.5** (2.1)
> 1.0- ≤ 1.5 Hr Post-HD	0.9 (1.7)	0.8 (1.6)	0.8 (2.9)
> 1.5- ≤ 2.0 Hr Post-HD	0.7 (1.5)	0.7 (1.4)	0.6 (1.7)
> 2.0- ≤ 6.5 Hr Post-HD	3.6 (7.4)	4.3 (6.1)	4.3 (9.2)
> 6.5- ≤ 11.0 Hr Post-HD	3.0 (7.0)	3.7 (4.5)	3.4 (7.0)
> 11.0- ≤ 15.5 Hr Post-HD	6.6 (13.7)	5.6 (15.4)	6.1 (13.7)
> 15.5- ≤ 20.0 Hr Post-HD	7.1 (8.3)	6.4 (12.0)	6.6 (8.8)
**Minutes of Light PA Per Predefined Granular Slice Period (*N*** **= 176)**			
**Slice Period during/after HD or Concurrent Time**	**HD Day (±SD)**	**1st Non-HD Day (±SD)**	**2nd Non-HD Day (±SD)**
	***0-to- ≤ 24 Hrs after Start HD***	***> 24-to- ≤ 48 Hrs after Start HD***	***> 48-to- ≤ 72 Hrs after Start HD***
HD Period	13.3 (18.0)	101.4*** (41.6)	95.7*** (50.6)
0.0- ≤ 0.5 Hr Post-HD	13.6 (5.5)	13.9 (7.3)	13.1 (8.6)
> 0.5- ≤ 1.0 Hr Post-HD	17.3 (6.1)	13.5*** (7.1)	13.5*** (8.7)
> 1.0- ≤ 1.5 Hr Post-HD	16.1 (6.9)	12.9*** (6.6)	12.9*** (9.2)
> 1.5- ≤ 2.0 Hr Post-HD	13.4 (6.5)	12.5 (6.9)	13.0 (9.9)
> 2.0- ≤ 6.5 Hr Post-HD	72.5 (43.6)	86.4** (53.7)	82.5 (54.3)
> 6.5- ≤ 11.0 Hr Post-HD	56.7 (45.2)	60.8 (47.8)	61.9 (49.2)
> 11.0- ≤ 15.5 Hr Post-HD	56.4 (50.7)	54.4 (48.0)	63.1 (57.2)
> 15.5- ≤ 20.0 Hr Post-HD	89.6 (56.9)	92.1 (42.6)	94.7 (53.3)
**Minutes of Sedentary Time Per Predefined Granular Slice Period (*N*** **= 176)**			
**Slice Period during/after HD or Concurrent Time**	**HD Day (±SD)**	**1st Non-HD Day (±SD)**	**2nd Non-HD Day (±SD)**
	***0-to- ≤ 24 Hrs after Start HD***	***> 24-to- ≤ 48 Hrs after Start HD***	***> 48-to- ≤ 72 Hrs after Start HD***
HD Period	178.0 (57.2)	98.8*** (49.6)	113.2*** (61.4)
0.0- ≤ 0.5 Hr Post-HD	14.6 (6.0)	14.6 (7.7)	15.9 (9.1)
> 0.5- ≤ 1.0 Hr Post-HD	11.3 (6.0)	14.8*** (7.7)	15.6*** (9.0)
> 1.0- ≤ 1.5 Hr Post-HD	12.7 (7.3)	15.6*** (7.1)	16.0*** (9.7)
> 1.5- ≤ 2.0 Hr Post-HD	14.8 (6.7)	15.5 (7.2)	15.7 (10.2)
> 2.0- ≤ 6.5 Hr Post-HD	125.3 (59.4)	118.3 (56.5)	128.9 (64.1)
> 6.5- ≤ 11.0 Hr Post-HD	92.6 (66.1)	91.2 (59.4)	101.9 (76.4)
> 11.0- ≤ 15.5 Hr Post-HD	64.9 (47.4)	61.9 (45.2)	75.6 (65.7)
> 15.5- ≤ 20.0 Hr Post-HD	82.6 (52.7)	80.9 (43.5)	90.3 (63.9)

***, *p* < 0.001; **, *p* < 0.01; *, *p* < 0.05

At concurrent times to HD on the subsequent first non-HD day, patients performed an average of 1308 more steps (*p* < 0.001), 0.1 higher MET (*p* < 0.001), 7.2 more minutes of MVPA (*p* < 0.001), 88.1 more minutes of light PA (*p* < 0.001), and 79.2 fewer minutes of sedentary time; on the second non-HD day patients performed 1128 more steps (*p* < 0.001), 0.1 higher MET (*p* < 0.001), 5.5 more minutes of MVPA (*p* < 0.001), 82.4 more minutes of light PA (*p* < 0.001), and 64.8 fewer minutes of sedentary time compared to HD days (Table 3). During HD patients exhibited MET levels indicative of sitting still/resting (MET = 1.0) and had 178.0 min of sedentary time, however they did perform low levels of some types of PA (97 steps, 0.3 min of MVPA, 13.3 min of light PA) that are likely attributable to slight movements during HD and could also be due to discrepancies in self-reported dialysis times captured from patient diaries.

Patients performed slightly more PA during the 2-h post-HD period on HD days versus concurrent times on subsequent non-HD days. During the 0-to-30, 31-to-60, and 61-to-90-min post-HD slices on HD days, patients tended to take more steps, perform more MVPA/light PA, and spend less time in a sedentary state compared to concurrent times on both the first and second non-HD days. During the 91-to-120-min slice on HD days, patients tended to perform slightly more steps than concurrent times on the first non-HD days (Table 3). There was a small signal for patients performing lower PA during the > 2-to- ≤ 6.5-h post-HD period on HD days versus the first non-HD days, where patients performed 13.9 fewer minutes of light PA (*p* = 0.009).

### Rates of granular physical activity

**Fig. 3:**
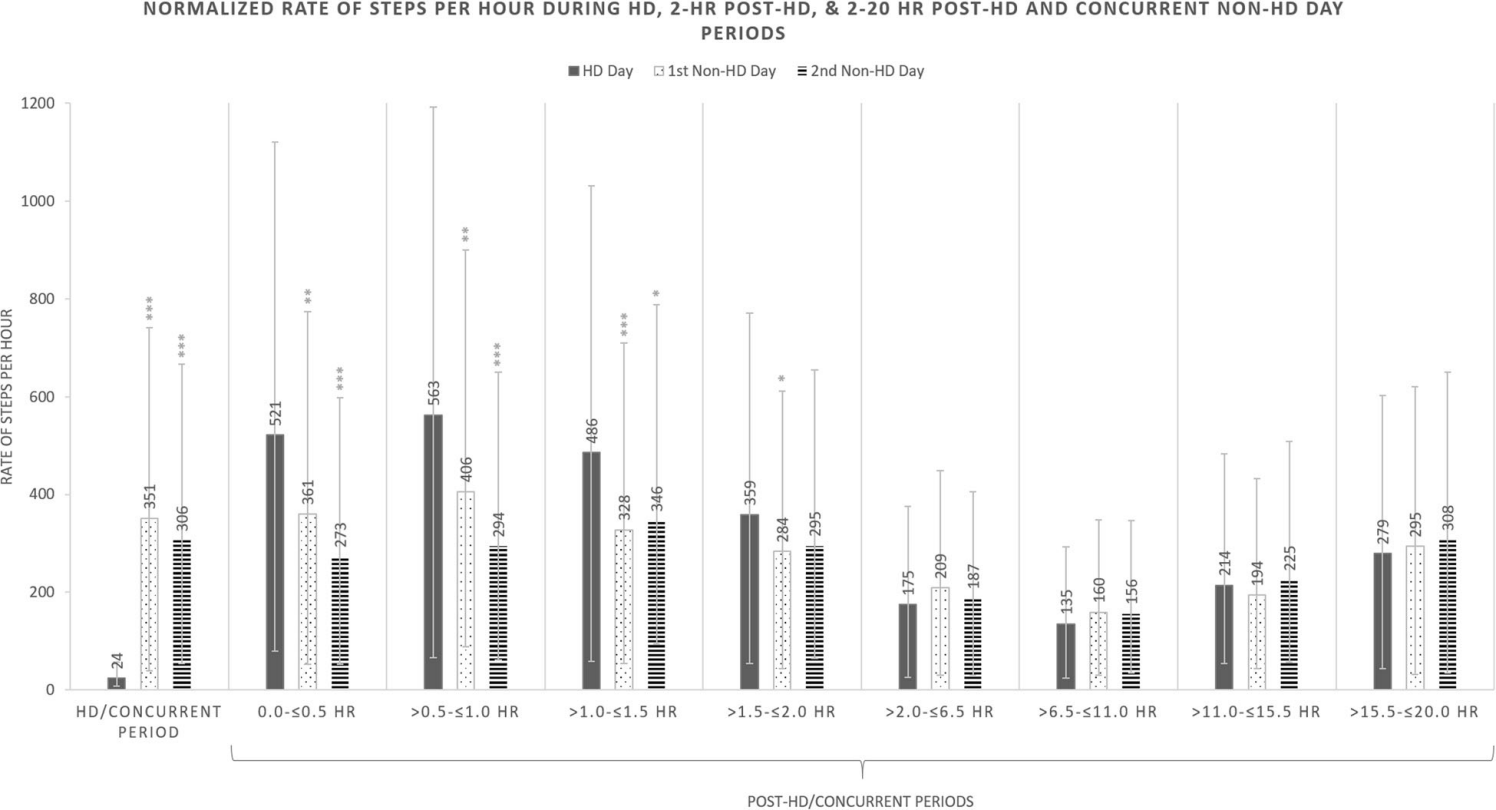
Normalized rates for steps per hour with 95% confidence intervals in the prespecified periods 0-to- ≤ 24 h after the start of HD on HD days (solid gray columns), > 24-to- ≤ 48 h after the start of HD on the first non-HD days (white with black doted columns), and > 48-to- ≤ 72 h after the start of HD on the second non-HD day (horizontal black striped columns). Times during and post-HD/concurrent periods are presented on the x axis and the rate of steps per hour is shown on the y-axis

**Fig. 4:**
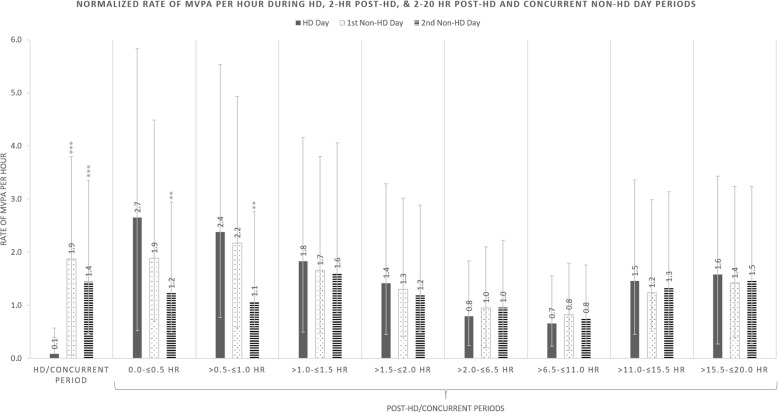
Normalized rates for minutes of MVPA per hour with 95% confidence intervals in the prespecified periods 0-to- ≤ 24 h after the start of HD on HD days (solid gray columns), > 24-to- ≤ 48 h after the start of HD on the first non-HD days (white with black doted columns), and > 48-to- ≤ 72 h after the start of HD on the second non-HD day (horizontal black striped columns). Times during and post-HD/concurrent periods are presented on the x axis and the rate of MVPA per hour is shown on the y-axis

Normalized rates of steps and minutes of MVPA performed per hour were calculated to visualize relative distinctions in prespecified periods (Figs. 3 and 4). Compared to the treatment time on HD days, patients performed rates of 327 and 282 more steps/hour and 1.8 and 1.4 more minutes of MVPA/hour during the concurrent times on the subsequent first and second non-HD days respectively (all *p* < 0.001).

Patients generally performed higher rates of steps and minutes of MVPA in the 2-h post-HD period on HD days (Figs. 3 and 4). In the 0-to-30-min post-HD period on HD days, patients performed rates of 161 and 248 more steps/hour compared to the first and second non-HD days respectively (both *p* < 0.01); patients performed 1.4 more minutes of MVPA/hour on HD days versus the second-non-HD day (*p* < 0.01). During the 31-to-60, 61-to-90, and 91-to-120-min slices of PA following HD, patients performed significantly more steps than concurrent times on both the first and second non-HD day (Fig. 3).

### Granular profiles of physical activity by transportation type

An exploratory sub-group assessment of granular PA levels for step counts by categories of transportation type on HD days found patients using public transportation (category including public transportation or walking) performed on average a sum of 604 more steps during the 2-h post-HD period versus patients using car transportation (category including family car, taxi, or ambulance) (Table 4). Patients using public transportation had on average 3.1 fewer minutes of sedentary time during only the predefined period > 1.5-to- ≤ 2 h after HD compared to patients using car transportation. No other differences were observed by transportation type for step counts and sedentary time in other prespecified timepoints in the 24 h during and after HD.

**Table 4:** Average Physical Activity (PA) Levels Per Predefined Granular Slice Period on HD Days by Transportation Type

Step Counts Per Predefined Granular Slice Period			
Slice Period during/after HD	***Steps on HD Days (±SD) with Public Transportation (N = 66)***	***Steps on HD Days (±SD) with Car Transportation (N = 110)***	***P-Value*** vs ***Car***
HD Period	128 (323)	78 (136)	0.156
0.0- ≤ 0.5 Hr Post-HD	364 (355)	201 (174)	< 0.0001
> 0.5- ≤ 1.0 Hr Post-HD	400 (269)	210 (154)	< 0.0001
> 1.0- ≤ 1.5 Hr Post-HD	320 (234)	197 (161)	< 0.0001
> 1.5- ≤ 2.0 Hr Post-HD	259 (218)	132 (135)	< 0.0001
> 2.0- ≤ 6.5 Hr Post-HD	880 (660)	732 (830)	0.227
> 6.5- ≤ 11.0 Hr Post-HD	639 (714)	589 (740)	0.679
> 11.0- ≤ 15.5 Hr Post-HD	1227 (2006)	808 (1419)	0.155
> 15.5- ≤ 20.0 Hr Post-HD	1502 (1524)	1113 (1227)	0.094
**Minutes of Sedentary Time Per Predefined Granular Slice Period**			
**Slice Period during/after HD**	***Sedentary Minutes on HD Days (±SD) with Public Transportation (N = 66)***	***Sedentary Minutes on HD Days (±SD) with Car Transportation (N = 110)***	***P-Value*vs*Car***
HD Period	186.1 (53.2)	173.1 (59.2)	0.147
0.0- ≤ 0.5 Hr Post-HD	13.6 (5.1)	15.1 (6.4)	0.101
> 0.5- ≤ 1.0 Hr Post-HD	11.7 (5.7)	11.0 (6.2)	0.473
> 1.0- ≤ 1.5 Hr Post-HD	11.8 (6.6)	13.3 (7.7)	0.185
> 1.5- ≤ 2.0 Hr Post-HD	12.8 (6.2)	15.9 (6.8)	0.003
> 2.0- ≤ 6.5 Hr Post-HD	119.3 (54.5)	128.9 (62.2)	0.307
> 6.5- ≤ 11.0 Hr Post-HD	86.6 (63.5)	96.3 (67.7)	0.379
> 11.0- ≤ 15.5 Hr Post-HD	68.8 (44.4)	62.6 (49.1)	0.457
> 15.5- ≤ 20.0 Hr Post-HD	76.5 (50.2)	86.2 (54.0)	0.284

Car transportation category includes transportation types of family car, taxi, and ambulance. Public transportation category includes transportation types of public transportation and walking

### Profiles of physical activity by Dialysis shift and day of the week

Patients with HD sessions starting before 1500 h (first and second HD shift) performed 2157 fewer steps (*p* < 0.0001), 18.6 fewer minutes of MVPA (*p* < 0.0001), and had 65.8 more minutes of sedentary time (*p* < 0.0001) per 24-h on HD days versus patients with HD sessions starting after 1500 h (third HD shift) (Table 5).

**Table 5:** Average Physical Activity (PA) Levels on HD Days Per 24 Hours by Dialysis Shift

PA Measure Per 24 Hrs on HD Days	HD start before 1500 Hrs (***N*** = 132)	HD start after 1500 Hrs (***N*** = 48)	***P***-Value vs HD before 1500 Hrs
Step Counts (±SD)	3390 (2313)	5547 (4613)	< 0.0001
Minutes of MVPA (±SD)	15.6 (20.0)	34.2 (46.2)	< 0.0001
Minutes of Sedentary Time (±SD)	538.5 (147.5)	472.7 (147.0)	< 0.0001

PA levels on HD days by shift calculated based on each HD start time before or after 1500 h. Among the 176 patients with accelerometry data, 4 patients received an HD treatment in both shifts during the 7 days of accelerometry

An exploratory sub-group analysis of step counts on HD days by shift and age category showed patients starting HD before 1500 h who were < 65 years old performed 1668 more steps per 24 h after HD compared to patients ≥65 years old (*p* < 0.0001), yet there were no significant distinctions in sedentary time (Table 6). There were no significant differences between step counts and sedentary time by age categories in patients with HD starting after 1500 h.

**Table 6:** Average Physical Activity (PA) Levels on HD Days Per 24 Hours by Dialysis Shift, Age, and Family Income

HD Shift	PA Measure Per 24 Hrs on HD Days	Age < 65 Years (***N*** = 130) Mean (±SD) N	Age ≥ 65 Years (***N*** = 46) Mean (±SD) N	***P***-Value
HD start before 1500 Hrs (*N* = 132)	*Steps*	3897 (2356) 92	2229 (1727) 40	< 0.0001
	*Sedentary Time*	530.9 (148.7) 92	555.9 (144.0) 40	0.127
HD start after 1500 Hrs (*N* = 48)	*Steps*	5732 (4134) 42	4413 (6892) 7	0.237
	*Sedentary Time*	463.0 (140.9) 42	531.7 (172.2) 7	0.053
HD Shift	**PA Measure Per 24 Hrs on HD Days**	**Family Income > 2 Minimum Wages (*N*** **= 142)** **Mean (±SD) N**	**Family Income < 2 Minimum Wages (*N*** **= 34)** **Mean (±SD) N**	***P*-Value**
HD start before 1500 Hrs (*N* = 132)	*Steps*	3326 (2296) 108	3673 (2386) 24	0.255
	*Sedentary Time*	546.3 (152.1) 108	504.1 (120.4) 24	0.029
HD start after 1500 Hrs (*N* = 48)	*Steps*	4620 (3127) 39	8491 (6891) 10	< 0.0001
	*Sedentary Time*	487.7 (119.9) 39	425.1 (206.3) 10	0.030

PA levels on HD days by shift calculated based on each HD start time before or after 1500 h. Among the 176 patients with accelerometry data, some patients stratified by age and income category received an HD treatment in both shifts during the 7 days of accelerometry

Another sub-group analysis of PA per 24 h on HD days by shift and family income level found no differences in step counts among patients starting HD before 1500 h with a family income above and below 2 minimum wages. However, patients starting HD before 1500 h with a family income < 2 minimum wages had 42.2 fewer minutes of sedentary time per 24 h after HD as compared to patients with a family income > 2 minimum wages (*p* = 0.029) (Table 6). Among patients with HD starting after 1500 h, those with a family income < 2 minimum wages took 3872 more steps (*p* < 0.0001) and had 62.6 fewer minutes of sedentary time (*p* = 0.030) per 24 h after HD versus patients with a family income > 2 minimum wages.

Granular assessment of PA identified patients with HD starting before 1500 h performed 116, 1741, and 871 fewer steps during the 0-to-30-min, > 11.0-to- ≤ 15.5-h, and > 15.5-to- ≤ 20.0-h post-HD periods on HD days respectively versus patients with HD starting after 1500 h (all *p* < 0.05) (Table 7). Despite this, patients with HD starting before 1500 h performed 68 and 680 more steps during the 91-to-120-min and > 2.0-to- ≤ 6.5-h post-HD periods versus patients with HD starting after 1500 h (both *p* < 0.05). Similar patterns of PA were observed for MVPA. Minutes of sedentary time during the periods 2-h post-HD did not differ between patients starting HD before versus after 1500 h. Patients starting before 1500 h had more minutes of sedentary time in the > 2.0-to- ≤ 11.0 h periods after dialysis HD (both *p* < 0.0001) and less minutes of sedentary time in the > 11.0-to- ≤ 20.0 h periods post-HD (both *p* < 0.05) compared to those starting HD after 1500 h.

**Table 7:** Average Physical Activity (PA) Levels on HD Days Per Predefined Granular Slice Period by Dialysis Shift

Step Counts Per Predefined Granular Slice Period (***N*** = 176)			
Slice Period during/after HD	HD Start before 1500 Hrs (±SD) (***N*** = 132)	HD Start after 1500 Hrs (±SD) (***N*** = 48)	***P***-Value vs before 1500 Hrs
HD Period	93 (244)	124 (254)	0.447
0.0- ≤ 0.5 Hr Post-HD	229 (231)	345 (347)	0.011
> 0.5- ≤ 1.0 Hr Post-HD	288 (231)	247 (191)	0.270
> 1.0- ≤ 1.5 Hr Post-HD	254 (194)	202 (210)	0.119
> 1.5- ≤ 2.0 Hr Post-HD	194 (179)	126 (176)	0.025
> 2.0- ≤ 6.5 Hr Post-HD	949 (798)	269 (319)	< 0.0001
> 6.5- ≤ 11.0 Hr Post-HD	570 (660)	767 (968)	0.192
> 11.0- ≤ 15.5 Hr Post-HD	344 (483)	2085 (2320)	< 0.0001
> 15.5- ≤ 20.0 Hr Post-HD	968 (1130)	1839 (1563)	< 0.0001
**MVPA Per Predefined Granular Slice Period (*N*** **= 176)**			
**Slice Period during/after HD**	**HD Start before 1500 Hrs (±SD) (*N*** **= 132)**	**HD Start after 1500 Hrs (±SD) (*N*** **= 48)**	***P*-Value vs before 1500 Hrs**
HD Period	0.3 (1.7)	0.5 (1.7)	0.605
0.0- ≤ 0.5 Hr Post-HD	1.0 (2.0)	2.2 (3.2)	0.005
> 0.5- ≤ 1.0 Hr Post-HD	1.2 (2.0)	1.1 (1.7)	0.747
> 1.0- ≤ 1.5 Hr Post-HD	0.9 (1.6)	1.0 (1.6)	0.773
> 1.5- ≤ 2.0 Hr Post-HD	0.7 (1.4)	0.6 (1.3)	0.4841
> 2.0- ≤ 6.5 Hr Post-HD	4.3 (6.7)	1.2 (2.0)	< 0.0001
> 6.5- ≤ 11.0 Hr Post-HD	2.7 (4.0)	4.5 (6.2)	0.059
> 11.0- ≤ 15.5 Hr Post-HD	2.2 (4.6)	14.2 (23.0)	< 0.0001
> 15.5- ≤ 20.0 Hr Post-HD	5.1 (8.6)	11.0 (16.2)	0.004
**Minutes of Sedentary Per Predefined Granular Slice Period (*N*** **= 176)**			
**Slice Period during/after HD**	**HD Start before 1500 Hrs (±SD) (*N*** **= 132)**	**HD Start after 1500 Hrs (±SD) (*N*** **= 48)**	***P*-Value vs before 1500 Hrs**
HD Period	174.6 (59.0)	192.6 (52.3)	0.062
0.0- ≤ 0.5 Hr Post-HD	14.9 (6.3)	13.5 (5.9)	0.170
> 0.5- ≤ 1.0 Hr Post-HD	11.2 (6.2)	11.1 (5.6)	0.944
> 1.0- ≤ 1.5 Hr Post-HD	12.1 (7.4)	14.6 (7.0)	0.047
> 1.5- ≤ 2.0 Hr Post-HD	14.6 (6.6)	16.0 (7.4)	0.222
> 2.0- ≤ 6.5 Hr Post-HD	150.4 (45.7)	50.3 (36.2)	< 0.0001
> 6.5- ≤ 11.0 Hr Post-HD	106.1 (62.1)	34.9 (50.0)	< 0.0001
> 11.0- ≤ 15.5 Hr Post-HD	57.9 (51.6)	79.7 (39.9)	0.011
> 15.5- ≤ 20.0 Hr Post-HD	67.7 (49.0)	114.4 (45.5)	< 0.0001

PA levels on HD days by shift calculated based on each HD start time before or after 1500 h. Among the 176 patients with accelerometry data, 4 patients received an HD treatment in both shifts during the 7 days of accelerometry

PA levels on HD days were similar on the first (Monday/Tuesday), second (Wednesday/Thursday) or third (Friday/Saturday) HD session of the week and did not differ between groups in ANOVA and t-test comparisons (Table 8).

**Table 8:** Average Physical Activity (PA) Levels on HD Days Per 24 Hours by Dialysis Day of the Week

HD Session of Week (***N*** = 176)	Steps Count Per 24 Hrs (±SD)	***P***-Value vs HD 1	Minutes of MVPA Per 24 Hrs (±SD)	***P***-Value vs HD 1
HD 1 (Monday/Tuesday)	3981 (3096)	Reference	21.0 (27.9)	Reference
HD 2 (Wednesday/Thursday)	3976 (3289)	0.987	20.5 (30.5)	0.872
HD 3 (Friday/Saturday)	3820 (3270)	0.637	19.1 (32.5)	0.560

Granular steps did not differ by day of the week. Patients performed 0.4 more minutes of MVPA during the 91-to-120-min post-HD period on the first versus the third HD session of the week (*p* = 0.042); no other differences were observed (Table 9).

**Table 9:** Average Physical Activity (PA) Levels on HD Days Per Predefined Granular Slice Period by Dialysis Day of the Week

Step Counts Per Predefined Granular Slice Period (***N*** = 176)					
Slice Period during/after HD	HD 1 (±SD)	HD 2 (±SD)	***P***-Value vs HD 1	HD 3 (±SD)	***P***-Value vs HD 1
HD Period	70 (142)	133 (550)	0.155	83 (183)	0.460
0.0- ≤ 0.5 Hr Post-HD	255 (300)	273 (311)	0.586	265 (337)	0.766
> 0.5- ≤ 1.0 Hr Post-HD	272 (294)	287 (277)	0.633	293 (336)	0.945
> 1.0- ≤ 1.5 Hr Post-HD	243 (246)	252 (252)	0.742	247 (256)	0.885
> 1.5- ≤ 2.0 Hr Post-HD	205 (247)	191 (278)	0.629	170 (203)	0.170
> 2.0- ≤ 6.5 Hr Post-HD	808 (814)	816 (1002)	0.936	858 (1137)	0.655
> 6.5- ≤ 11.0 Hr Post-HD	568 (758)	675 (1087)	0.363	645 (774)	0.426
> 11.0- ≤ 15.5 Hr Post-HD	1132 (2038)	1081 (2078)	0.851	1047 (1971)	0.752
> 15.5- ≤ 20.0 Hr Post-HD	1441 (1588)	1337 (1585)	0.598	1253 (1649)	0.355
**MVPA Per Predefined Granular Slice Period (*N*** **= 176)**					
**Slice Period during/after HD**	**HD 1 (±SD)**	**HD 2 (±SD)**	***P*-Value vs HD 1**	**HD 3 (±SD)**	***P*-Value vs HD 1**
HD Period	0.4 (1.2)	0.5 (4.3)	0.656	0.2 (0.9)	0.059
0.0- ≤ 0.5 Hr Post-HD	1.4 (2.8)	1.3 (2.9)	0.665	1.3 (2.9)	0.642
> 0.5- ≤ 1.0 Hr Post-HD	1.2 (2.5)	1.2 (2.5)	0.551	1.1 (3.1)	0.762
> 1.0- ≤ 1.5 Hr Post-HD	1.0 (2.3)	0.9 (1.8)	0.570	0.9 (2.0)	0.520
> 1.5- ≤ 2.0 Hr Post-HD	0.9 (2.2)	0.8 (2.3)	0.840	0.5 (1.3)	0.042
> 2.0- ≤ 6.5 Hr Post-HD	3.5 (6.4)	3.4 (7.9)	0.970	4.4 (11.2)	0.336
> 6.5- ≤ 11.0 Hr Post-HD	2.9 (4.9)	2.9 (6.3)	0.944	3.4 (6.5)	0.501
> 11.0- ≤ 15.5 Hr Post-HD	7.5 (19.1)	7.5 (18.5)	0.998	7.2 (19.8)	0.905
> 15.5- ≤ 20.0 Hr Post-HD	8.1 (14.1)	8.2 (16.6)	0.947	6.2 (14.4)	0.275

## Discussion

This analysis of baseline data from the HDFIT trial characterized the profiles of PA in reference to the timing of HD and revealed unique PA patterns in the post-HD period, compared to concurrent times on subsequent non-HD days. Clinically stable prevalent HD patients in the first 2 years of HD who did not have limitations in ambulation tended to be highly sedentary, with levels of PA that only increased their metabolic energy expenditure rate by one tenth of a kcal/kg/hour above the resting level. As anticipated, PA levels per 24 h were lower on HD days compared to the first and second non-HD days. PA levels and patterns per 24 h are consistent with other literature reports of PA measured on calendar days (i.e. 0:00–23:59 h) [7, 18–25]. Specialized accelerometry methods allowed us to characterize PA relative to the time of the start of HD. Lower PA levels on HD days were found to be primarily attributable to inactivity during HD, accounting for > 1000 fewer steps on HD days. Contrary to our main hypothesis, patients had higher levels and the highest rates of PA within 2 h after HD as compared to concurrent times on subsequent non-HD days, yet this only accounted for about 300 additional steps. PA profiles differed in later HD shifts, with HD staring after 1500 h being associated with patients taking > 2000 more steps 24 h after HD. These distinct levels were driven by activity performed within 30 min after HD and 11-to-20 h after HD. Although the findings might be representative of late shift patients being a more functional subgroup who perform more daily activities, age and family income level did not appear to be influencing the distinctions in PA levels between earlier and later HD shifts. The distinctions in PA performed 11-to-20 h after HD may also be attributed to the timing of sleep after HD in the later shift patients and PA patterns performed the subsequent day.

The literature reports relatively sedentary levels of PA in the HD population (< 5000 steps/day, < 21.5 min of MVPA/day, < 3.0 MET (kcal/kg/hour) or equivalent measures), however, large variations exist (differences up to 6000+ steps/day) depending on populations and geographies [1, 2, 6, 7, 18–25, 39]. Our findings are consistent with HD patients generally performing < 4000 steps per 24-h period on HD days, and about 5000 steps per 24-h period on non-HD days. Despite this, patients achieved goals for MVPA on non-HD days in line with WHO recommendations of ≥150 min of MVPA/week [39]. However, MET levels were only 0.1 kcal/kg/hour above the resting level.

Contrary to our expectation, PA was higher following HD. This is an unexpected finding given patient-reported dialysis recovery time is perceived to be longer than 2 h for more than 65% of patients and recovery time associates negatively with physical component summary scores of the Kidney Disease Quality of Life (KDQOL) survey [40]. However, consistent with our findings, a small accelerometry study using the RT-3 monitor (StayHealthy, Monrovia, CA) among 20 HD patients has previously found raw vector magnitude acceleration counts per minute (CPM) were significantly higher within the 2 h period following dialysis on HD days, as compared to the same time on non-HD days [41]. Despite that the accelerometer used was unable to compute step counts for generalizability, nor filter raw acceleration data, this study’s results and ours showed consistent signals of a potentially important timepoint for more PA being performed proximal to dialysis. Future investigations appear warranted to investigate potential inverse associations in patient perceptions of self-reported dialysis recovery time and measured PA post-HD. Also, given this RCT included a select population, further investigations are needed to confirm our findings are generalizable in the overall HD population.

Increased post-HD PA might be associated with transportation from the clinic, especially in the first hour when step counts and rates are the highest. Post-HD PA levels appear to be influenced by transportation type with patients using public transportation and walking performing about 600 more steps during the 2-h period after HD compared to those using of a family car, taxi, and ambulance; this finding coincides with no differences in sedentary time between groups up to 90 min after HD, and only 3 min less sedentary time in those using public transportation and walking in the prespecified period 90-to-120 min after HD. Other contributing factors may include improved fluid and electrolyte status after HD, and routine activities performed after HD (i.e. cooking, shopping, errands), which may associate to increased levels of light PA observed 31-to-90 min after HD. The finding that PA rates are the highest in the first hours after HD might be signaling an important timepoint in the post-HD period that warrants investigation in the future. It has been recognized sudden death typically occurs within 12 h of HD [42]. However, it is unknown if higher PA rates could cause hemodynamic stress immediately following HD, which itself inherently causes a hemodynamic challenge [43].

HD schedules before/after 1500 h might also influence PA on HD days. Compared to patients starting HD before 1500 h, those treated after 1500 h presented higher step counts at 30 min and 11-to-20 h after HD. Despite this, patients starting HD before 1500 h took more steps 1.5-to-6.5 h after HD compared to patients starting HD after 1500 h; relatively consistent signals were observed with MVPA and sedentary time. We found younger patients (< 65 years old) treated in earlier HD shifts performed > 1600 more steps per 24 h than patients ≥65 years old with no differences by age in sedentary time. However, step counts and sedentary time did not significantly differ by age in later HD shift patients. Although the income level was not associated with distinctions in step counts for earlier HD shift patients, those with a low family income that were treated in earlier shifts had about 40 fewer minutes of sedentary time per 24 h after HD. Among later shift patients, a low family income level was associated with patients taking about 3800 more steps and having about 60 less minutes of sedentary time per 24 h. Despite this, within sub-group differences for age and family income sub-groups were relatively consistent with changes in the overall cohort. Our findings could possibly be related to free living PA and sleep patterns, as well as other patient characteristics; a selection bias may be contributing to the findings. Patients treated on the first HD shift have been previously found to be associated to altered sleep patterns, [6, 7] and the patterns and timing of sleep between patients treated in earlier and later shifts may be reflected in the distinctions in PA found 90 min to 20 h after HD. The HD day of the week did not remarkably affect PA.

PA is of utmost importance to improving/maintaining health in all populations, and it is a useful surrogate marker of negative outcomes. The United States guidelines for PA suggest performing any higher quantity of PA yields some health benefits [44]. Select European and Oceania countries have adopted the concept that exercise is “medicine” for dialysis patients and should be prescribed, but unfortunately, this philosophy has not been adopted in many countries in the world [45]. General and elderly populations have been shown to exhibit an inverse relationship between steps taken and outcomes [46–50]. Achievement of increases of 1000 more steps per day associates with about a 5% lower cardiovascular event rate and a 15% lower all-cause mortality rate [46, 47]. A 7-year observational study in HD patients found taking ≥5000 steps a day associates with an 18.5 percentage point improvement in the cumulative survival rate compared to having < 5000 steps daily [24]. In addition, this study found patients who had a 30% decrease in daily steps had a 3-fold higher adjusted risk of death compared to patients with a 30% increase [24].

Intradialytic exercise programs could potentially increase PA profiles on HD days. We established patients perform the highest levels and rates of activity within 2 h of HD, the requirements of sitting/lying during the HD treatment was found to be the primary cause of higher inactivity on HD days, which is consistent with, confirms, and builds upon findings from a small prior accelerometry study [41]. Clinicians, care teams and the nephrology community should recognize HD regimens are a major factor negatively impacting activity levels. A RCT has shown 4 months of intradialytic pedaling exercise decreased arterial stiffness [51]. Although intradialytic exercise such as cycling/pedaling may be a low hanging fruit for targeting interventions, with a higher potential to yield beneficial health outcomes, it can only reach patients 12 h per week. Developing both intradialytic and interdialytic exercise programs should be a high priority for providers and clinicians as a means to increase PA in this largely sedentary population.

We believe it will be of importance for clinicians to prescribe exercise as a medicine in a patient centric manner and routinely track adherence. Programs can be simple and not require additional resources, such as prescribing a 20 min walk every day and leg lifts/stretching/Pilates during each HD treatment. It may be important to consider small incremental increases that focus on HD patients performing more activity, rather than achieving a specific exercise intensity. Our findings among patients without any impaired mobility/ambulation can be applied to the design of interventions aiming to increase PA in similar HD populations. They suggest the timing of interdialytic exercise on HD days might be most optimal to be recommended before or > 2 h after HD when PA levels tend to be lower. It appears relevant to tailor exercise recommendations based on transportation type, with an emphasis of efforts in patients using a car to come to/from the clinic. On days without HD, exercise could be encouraged at the patient’s discretion. Patients treated in earlier HD shifts are possibly an important sub-group of patients to target with exercise interventions. Among this sub-group, older patients performed the lowest PA, yet younger patients were also highly inactive taking < 4000 steps per 24 h. Therefore, age may not be an appropriate consideration in targeting exercise interventions if no other contraindications to performing PA exist.

While our study has many strengths including use of high-quality RCT data, there are some limitations. Patients may not be representative of the overall HD population, but specifically generalizable to patients in the first 2 years of HD without impaired mobility/ambulation who are adherent with HD. We used self-reported HD times which may be inaccurate. There is also a potential for undercounting of PA using ActiLife software default algorithm, which has been validated in younger people performing exercise [37, 38]. Nonetheless, accelerometry is internally consistent and PA levels were found to be similar with previous reports [7, 18–25]. Rates of PA were calculated by period without adjustment for sleep time, which was excluded per protocol. Although this does not influence absolute values of PA, it could affect normalized rates.

## Conclusions

In conclusion, the findings identify patients performed higher PA levels and the highest PA rates in the 2-h post-HD period on HD days compared to concurrent times on non-HD days, however, the requirement of sitting/lying during HD causes overall 24-h PA levels on HD days to be lower than non-HD days. Timing of the HD session associates to distinct PA levels, but further investigations are needed. Exercise programs should be developed that increase activity during and between HD treatments.

## Supplementary information

**Additional file 1: Appendix A.** HDFIT Study Site Investigators and Trial Leadership; **Appendix B.** EPICENTER ACRO HDFIT Key Leadership and Affiliates; **Appendix C.** HDFIT Steering Committee.

## Data Availability

The original source documents and electronic datasets, from the trial, as well as the coding from this analysis are not publicly available. The original trial documents, datasets, and coding are regulated and maintained by the EPICENTER ACRO. Affiliated investigators can access the trial dataset under oversight and approval of the HDFIT steering committee.

## Abbreviations

ACRO: Academic clinical research organization
ANOVA: Analysis of variance
BUN: Blood urea nitrogen
CKD: Chronic kidney disease
CPM: Counts per minute
DBP: Diastolic blood pressure
EPICENTER: Center for Epidemiology and Clinical Research
ESKD: End stage kidney disease
HD: Hemodialysis
HDF: Hemodiafiltration
iPTH: Intact parathyroid hormone
Kt/V: Dialysis adequacy
MET: Metabolic rate
MVPA: Moderate to vigorous physical activity
PA: Physical activity
PUCPR: Pontifícia Universidade Católica do Paraná
RCT: Randomized controlled trial
SBP: Systolic blood pressure
