# Supplementary material for: Impact of hemodialysis and post-dialysis period on granular activity levels
Source: BMC Nephrol. 2020 May 25;21:197. doi: 10.1186/s12882-020-01853-2 (PMC7249440; doi:10.1186/s12882-020-01853-2)
Supplement: Supplementary file 1 — Additional file 1: Appendix A. HDFIT Study Site Investigators and Trial Leadership; Appendix B. EPICENTER ACRO HDFIT Key Leadership and Affiliates; Appendix C. HDFIT Steering Committee. [file 12882_2020_1853_MOESM1_ESM.docx]

**Additional File 1**

**Appendices:**

| **Appendix A: HDFIT Study Site Investigators and Trial Leadership** | | |
| --- | --- | --- |
| **Site** | **Principal Investigator** | **Lead Coordinator** |
| Irmandade da Santa Casa de Misericórdia de Curitiba, Curitiba, Paraná | Ana Claudia Dambiski, MD | Thaylane Amanda de Souza, RN |
| Hospital de Clínicas - FMB - UNESP, Botucatu, São Paulo | Daniela Ponce, MD | Edwa Maria Bucuvic, RN |
| Nefron Contagem, Contagem, Minas Gerais | Luciana Menin Ferreira, MD | Wanderson de Souza Carvalho, RN |
| Clínica de Diálise Ingá, Rio de Janeiro, Rio de Janeiro | Jorge Paulo Strogoff de Matos, MD | Esther Oliveria Silva, RN |
| Instituto de Nefrologia de Taubaté, Taubaté, São Paulo | Manuel Carlos Martins de Castro, MD | Celina de Fátima e Silva, RN |
| Hospital do Rim e Universidade Federal de São Paulo, São Paulo, São Paulo | Maria Eugenia F Canziani, MD | Silvia R Manfredi, RN |
| Clínica de Doenças Renais – Botafogo, Rio de Janeiro, Rio de Janeiro | Katia Santos, MD | Ana Paula Fonseca Correia, RN |
| Hospital São Lucas PUCRS, Porto Alegre, Rio Grande do Sul | Giovani Gadonski, MD | Adriana Conti, RN |
| Clínica de Doenças Renais - São Lourenço, Rio de Janeiro, Rio de Janeiro | Inah Pecly, MD | Camille Souza Paixão, RN |
| Fundação Pró Rim, Joinville, Santa Catarina | Viviane Calice-Silva, MD, PhD | Simone Ribeiro, RN |
| Instituto Médico Nefrológico, Belo Horizonte, Minas Gerais | Lizia Regina Ribeiro Caldeira, MD | Adailto Santos, RN |
| CETENE, São Paulo, São Paulo | Rosilene Motta Elias, MD | Andreia Barbosa Dos Santos, RN |
| Hospital Alemão Oswaldo Cruz, São Paulo, São Paulo | Américo Lourenço Cuvello-Neto, MD | Amanda Monteiro Virolli, RN |

| **Appendix B: EPICENTER ACRO HDFIT Key Leadership and Affiliates** | | |
| --- | --- | --- |
| **Research Staff Member** | **Role** | **Affiliation** |
| Roberto Pecoits-Filho, MD, PhD, FASN, FACP | Principal Investigator & Director of Trial | Pontifícia Universidade Católica do Paraná |
| Juliane Woehl, PharmD | eCRF Designer, Trial & Data Manager | Pontifícia Universidade Católica do Paraná |
| Ludimila Guedim de Campos, PharmD | Project, Central Monitoring, & Regulatory Manager | Pontifícia Universidade Católica do Paraná |
| Priscila Bezerra Gonçalves, PhD | Lead Accelerometry Manager & Data Analyst | Pontifícia Universidade Católica do Paraná |
| Jochen G Raimann, MD, PhD, MPH | Lead Analytics Oversight & Trial Database Manager | Renal Research Institute |
| Murilo Guedes, MD, MS | Assistant Analytics Oversight, Data Analyst, & Trial Database Manager | Pontifícia Universidade Católica do Paraná |
| Shimul M Sheth | Data Analyst & Data Visualization Manager | Renal Research Institute |
| Hao Han, MS | Data Analyst & Lead Diary & Accelerometry Data Manager | Fresenius Medical Care North America |
| Sinaia Canhada, RN | Lead Field Monitor and Implementation Manager | Fresenius Medical Care Brazil |
| Andréa Novais Moreno Amaral, PhD | Lead Biorepository Specimen Manager | Pontifícia Universidade Católica do Paraná |
| Ana Clara Simões Flórido Almeida, MS | Assistant Project and Biorepository Specimen Manager | Pontifícia Universidade Católica do Paraná |
| Thyago Proença de Moraes, MD, PhD | Lead Analytical Design Manager | Pontifícia Universidade Católica do Paraná |
| Leticia Adejani Laibida, PhD | Lead Diary Data Manager | Pontifícia Universidade Católica do Paraná |
| Diane M Rondeau | Diary Data Analyst | Fresenius Medical Care North America |
| John Larkin, MSc, CCRC | Assistant Project and Data Mobility Manager | Pontifícia Universidade Católica do Paraná  Fresenius Medical Care North America |

| **Appendix C: HDFIT Steering Committee** | |
| --- | --- |
| **Member** | **Affiliation** |
| Maria Eugenia F. Canziani, MD | Universidade Federal de São Paulo |
| Roberto Pecoits-Filho, MD, PhD, FASN, FACP | Pontifícia Universidade Católica do Paraná |
| Carlos Eduardo Poli de Figueiredo, MD, PhD | Pontifícia Universidade Católica do Rio Grande do Sul |
| Américo Lourenço Cuvello Neto, MD | Hospital Alemão Oswaldo Cruz |
| Ana Beatriz Lesqueves Barra, MD, MBA | Fresenius Medical Care Brazil |
